# A machine-learning model to predict suicide risk in Japan based on national survey data

**DOI:** 10.3389/fpsyt.2022.918667

**Published:** 2022-08-04

**Authors:** Po-Han Chou, Shao-Cheng Wang, Chi-Shin Wu, Masaru Horikoshi, Masaya Ito

**Affiliations:** ^1^Department of Psychiatry, China Medical University Hsinchu Hospital, China Medical University, Hsinchu, Taiwan; ^2^Department of Psychiatry, China Medical University Hospital, China Medical University, Taichung, Taiwan; ^3^Department of Psychiatry, Taoyuan General Hospital, Ministry of Health and Welfare, Taoyuan, Taiwan; ^4^Department of Mental Health, Johns Hopkins Bloomberg School of Public Health, Baltimore, MD, United States; ^5^Department of Medical Laboratory Science and Biotechnology, Chung Hwa University of Medical Technology, Tainan, Taiwan; ^6^National Center for Geriatrics and Welfare Research, National Health Research Institutes, Zhunan Town, Yunlin County, Taiwan; ^7^Department of Psychiatry, National Taiwan University Hospital, Douliu, Taiwan; ^8^National Center for Cognitive-Behavior Therapy and Research, National Center of Neurology and Psychiatry, Tokyo, Japan

**Keywords:** machine learning, Super Learner, suicide, prediction model, suicide risk, Japan

## Abstract

**Objective:**

Several prognostic models of suicide risk have been published; however, few have been implemented in Japan using longitudinal cohort data. The aim of this study was to identify suicide risk factors for suicidal ideation in the Japanese population and to develop a machine-learning model to predict suicide risk in Japan.

**Materials and Methods:**

Data was obtained from Wave1 Time 1 (November 2016) and Time 2 (March 2017) of the National Survey for Stress and Health in Japan, were incorporated into a suicide risk prediction machine-learning model, trained using 65 items related to trauma and stress. The study included 3,090 and 2,163 survey respondents >18 years old at Time 1 and Time 2, respectively. The mean (standard deviation, SD) age was 44.9 (10.9) years at Time 1 and 46.0 (10.7) years at Time 2. We analyzed the participants with increased suicide risk at Time 2 survey. Model performance, including the area under the receiver operating characteristic curve (AUC), sensitivity, and specificity, were also analyzed.

**Results:**

The model showed a good performance (AUC = 0.830, 95% confidence interval = 0.795–0.866). Overall, the model achieved an accuracy of 78.8%, sensitivity of 75.4%, specificity of 80.4%, positive predictive value of 63.4%, and negative predictive value of 87.9%. The most important risk factor for suicide risk was the participants' Suicidal Ideation Attributes Scale score, followed by the Sheehan Disability Scale score, Patient Health Questionnaire-9 scores, Cross-Cutting Symptom Measure (CCSM-suicidal ideation domain, Dissociation Experience Scale score, history of self-harm, Generalized Anxiety Disorder-7 score, Post-Traumatic Stress Disorder check list-5 score, CCSM-dissociation domain, and Impact of Event Scale-Revised scores at Time 1.

**Conclusions:**

This prognostic study suggests the ability to identify patients at a high risk of suicide using an online survey method. In addition to confirming several well-known risk factors of suicide, new risk measures related to trauma and trauma-related experiences were also identified, which may help guide future clinical assessments and early intervention approaches.

## Introduction

Despite advances in the diagnosis and treatment of mental illness, suicide remains to be a major public health problem, with annual suicide rates at approximately 10–12 per 100,000 people over the past 60 years (1). Therefore, an increased understanding of the risk factors for suicide is important for early intervention and prevention. For the past 50 years, extensive work has been conducted to improve the prediction of suicide, yet a recent published meta-analysis demonstrated that using known suicide risk factors leads to modest results (weighted area under the receiver operating characteristic curve [AUC], 0.58) (2). Several factors may have led to this prediction failure (3). Firstly, suicidal rate in the population is relatively low, making prospective studies not practical (1). Second, prior studies were often limited to small samples, measured at a single time point, and examined few number of factors. Finally, the traditional method for statistical analysis of the suicide data mainly focus on inference, which resulted in simple prediction models; lastly, they are not designed to incorporate new clinical data to continuously update the existing models (3).

Recently, novel statistical analyses, such as machine learning, and big data sources, such as electronic health records or national survey data, have led to enormous improvements in predicting suicide risk in clinical practice (AUC, 0.63–0.94) (1, 4–7). However, most of the published work mainly focused on high-risk groups who sought for medical treatment (8). As it has been reported that more than one-third of the people attempting suicide do not actively seek medical treatment (9), it is essential to extend suicide prediction models beyond the treatment-seeking populations to the general population. Previous studies using population-based cohort data to build suicide prediction models have also yielded fair performance in general adult (10, 11) and adolescent (12) populations (AUC, 0.62–0.86). However, because of the variable suicide rates among countries with different cultural backgrounds, the suicide prediction model in one country may not be generalizable to another.

In view of the aforementioned limitations, we aimed to identify important risk factors for future suicide risk in a longitudinal cohort from the National Survey for Stress and Health (NSSH) dataset (13) in Japan, using an explanatory machine-learning model. This study aims to extend prior research in two directions. First, we used a large longitudinal sample to identify risk factors for suicidal ideation in the Japanese population. Second, we included an extensive assessment instrument that included detailed psychometric assessments for substance use, psychiatric disorders, personality traits, and clinical symptoms, which are not routinely available in electronic health records or administrative data. Overall, we expected to develop a model predicting suicide risk in a longitudinal cohort in Japan.

## Methods

### Database

The data of the present study were extracted from the National Survey for Stress and Health (NSSH), conducted between 2016 and 2017. Detailed information on NSSH can be found in our previous work (13–15). In brief, two waves of surveys were conducted. Wave 1 (*n* = 3,090) consisted of screening (November 2016), Time 1 (November 2016), and Time 2 surveys (March 2017). Wave 2 (*n* = 3,090) consisted of screening and the Time 1 survey (both in March 2017) (15). Recruitment emails were sent to 100,077 panelists in November (Wave 1). The target sample size in our study was 6,000 individuals, including 3,000 patients who met the probable diagnostic criteria based on the Diagnostic and Statistical Manual of Mental Disorders, 5th Edition (DSM-5) for posttraumatic stress disorder (PTSD) using the PCL-5, 1,000 non-clinical responders denting any past traumatic experience, and 2,000 non-clinical or subclinical responders with traumatic experiences. We terminated the screening when reaching half of the target sample size (i.e., 3,000 participants). The screened participants answered questions measuring their psychiatric symptoms and psychological processes at Times 1 and 2. Only participants at Wave 1 participated the Time 2 survey, which was conducted 4 months after Time 1.

All participants had read a full explanation of the research project and gave informed consent before answering the questionnaires. All survey contents were examined with design, logical flow, validity, and checking for errors by nine experienced psychologists and double-checked by two macromill survey engineers. To improve the data quality, the online survey system automatically excluded responders who answers the questions rapidly. Because the survey was designed to not allow participants to proceed if there are unanswered items, no data were missing except for income. This study was approved by the Institutional Review Board of the National Center of Neurology and Psychiatry (approval number: A2015-086).

### Participants

This study used longitudinal data collected in Wave 1, including Time 1 survey data (*n* = 3,090) and self-reported suicide ideation at the follow-up 4 months later (Time 2, *n* = 2,163). The cumulative response rate for Wave 2 was 66.7 %.

### Assessment of risk factors at time 1

#### Demographics

Personal information, including sex, age, income, marital status, substance use, history of physical or psychological abuse, or self-harm behavior; diagnosis and treatment for any psychiatric disorder, including major depressive disorder (MDD), bipolar disorder, dysthymic disorder, seasonal affective disorder, obsessive compulsive disorder, panic disorder, PTSD, generalized anxiety disorder, psychotic disorder, and eating disorder were recorded (Table 1).

**Table 1:** Characteristics of study participants.

**Characteristic**	**Participants of wave 1**	**Wave 1 participants** **Follow-ups**
	*N* = 3090	*N* = 2163
Female	1,509 (48.8%)	990 (45.8%)
Age (mean ±SD)	44.9 ± 10.9	46 ± 10.7
Marital status		
Married	1,466 (47.4%)	1,038 (48.0%)
Personal yearly income (Japanese yen)^a^		
0–1,999,999	1,460 (54.3%)	1,019 (49.7%)
2,000,000–3,999,999	572 (21.3%)	420 (20.5%)
4,000,000–5,999,999	320 (11.9%)	237 (11.6%)
6,000,000–7,999,999	197 (7.3%)	140 (6.8%)
8,000,000–9,999,999	80 (3.0%)	60 (2.9%)
Over 10,000,000	40 (1.5%)	36 (1.7%)
Hx of physical abuse	1,222 (39.6%)	818 (37.8%)
Hx of emotional abuse	1,875 (60.7%)	1,284 (59.4%)
Hx of self-harm behavior	778 (25.2%)	539 (24.9%)
Psychiatric comorbidities diagnosed and treated in medical settings		
MDD	1,630 (52.8%)	1,159 (53.6%)
Bipolar disorder	335 (10.8%)	232 (10.7%)
Dysthymic disorder	341 (11.0%)	234 (10.8%)
SAD	489 (15.8%)	350 (16.2%)
GAD	487 (15.8%)	350 (16.2%)
Panic disorder	630 (20.4%)	434 (20.0%)
OCD	471 (15.2%)	332 (15.4%)
PTSD	422 (13.7%)	289 (13.4%)
Psychosis	321 (10.4%)	210 (9.7%)
Eating disorder	293 (9.5%)	202 (9.3%)
SIDAS score at Time 1	20.0 +/– 9.7	19.8 +/– 9.5
SIDAS score at Time 2		19.6 +/– 9.1

a *1 Japanese yen is approximately equal to 0.0074 US dollar.*

*SD, standard deviation; MDD, major depressive disorder; SAD, seasonal affective disorder; GAD, generalized anxiety disorder; OCD, obsessive compulsive disorder; PTSD, post-traumatic stress disorder; SIDAS, suicidal ideation attributes scale*.

### Measures

#### PCL-5

We used the Japanese version of the PCL-5 to assess PTSD symptoms of the responders. The PCL-5 comprises a 20-item assessment, available from the National Center for PTSD (13). The 20 items are concordant with the DSM-5 diagnostic items for PTSD. Each question were answered with a 5-point Likert scale (0 = not at all, 1 = a little bit, 2 = moderately, 3 = quite a bit, 4 = extremely).

#### Trauma-related guilt inventory (TRGI)

The TRGI was developed by Kubany to assess the emotional and cognitive aspects of guilt associated with a specific traumatic event (16). The final version consists of 32 items on six scales: the Guilt Cognition Scale (which comprises three empirically derived subscales: Hindsight-Bias/Responsibility (seven items), Wrongdoing (five items), and Insufficient Justification (four items), along with an additional six general cognition items), the Distress Scale (six items), and the Global Guilt Scale (four items). The answers for all 32 items were recorded on a 5-point scale, with poles from “extremely true/always true” to “not at all true/never true” (eight items were reverse-scored).

#### Impact of event scale-revised (IES-R)

The Impact of Event Scale-revised (IES-R) is a widely used self-report measure in the field of traumatic stress (17). It contains 22 questions used to assess the core psychological phenomena of traumatic stress: intrusion (eight questions), avoidance (eight questions), and hyperarousal (six questions). A scoring scheme with intervals of 0, 1, 2, 3, and 5 was adopted for responders regarding the degree to which they were distressed or bothered by the listed conditions in the past 7 days from “not at all,” “a little bit,” “moderately,” “quite a bit,” to “extremely.”

#### Patient health questionnaire-9 (PHQ-9)

The PHQ-9 is a nine-item assessment for depressive symptoms experienced for the past 2 weeks (18). Responses were rated on a 4-point Likert scale (0 = not at all, 3 = nearly every day). The reliability and validity of PHQ-9 have been established in previous studies.

#### Generalized anxiety disorder 7-item scale (GAD-7)

The GAD-7 assesses symptoms of generalized anxiety experienced over the past 2 weeks (19). A seven-item questionnaire was developed that asked participants how often they were bothered by the listed anxiety symptoms during the past 2 weeks. The response options were “not at all,” “several days,” “more than half the days,” and “nearly every day,” scored as 0, 1, 2, and 3, accordingly. Scores of 5, 10, and 15 were used as the cutoff points for mild, moderate, and severe anxiety, respectively.

#### Sheehan disability scale (SDS)

The SDS is a three-item assessment for functional impairment in three domains: work/school, social life, and family life/home responsibility (20); higher scores imply more severe functional impairment.

#### Cut-annoyed-guilty-eye (CAGE) questionnaire

The CAGE is used for brief assessment of alcoholism (21). This questionnaire comprises four items: desire to reduce drinking, annoyance at being criticized for drinking, feeling guilty about drinking, and drinking in the morning to wake up. Participants responded with yes/no answers.

#### Tobacco dependence screener (TDS)

The TDS is a ten-item questionnaire for screening tobacco dependence, as defined by the Tenth revision of the International Statistical Classification of Diseases and Related Health Problems, DSM-III-Revision, and DSM-IV (22). The participants provided yes/no answers on each item.

#### Patient-reported version of the level 1 cross-cutting symptom measure for the DSM-5 (CCSM)

The Level 1 CCSM is a 23-item assessment of 13 domains of symptoms common to psychiatric disorders (23). Test-retest reliability for each domain was fair in a DSM-5 field trial.

#### Eysenck personality questionnaire revised-short form (EPQR-S)

The EPQR-S is a self-report questionnaire consisting of 48 items, 12 for each trait of neuroticism, extraversion, and psychoticism, and 12 on the lie scale. Each question has a binary “yes” or “no” response. The dichotomous item is scored as 1 or 0, and each scale has a maximum score of 12 and a minimum of zero (24).

#### Posttraumatic maladaptive beliefs scale (PMBS)

The PMBS is a 15-item scale developed to measure maladaptive beliefs about current life circumstances following trauma exposure (25). This scale assesses maladaptive beliefs in three domains: (a) threat of harm, (b) self-worth and judgment, and (c) reliability and trustworthiness of others. Each item included in the PMBS was rated using a 7-point Likert-type response format, ranging from one (not at all true) to seven (completely true). A list of subscale items and reverse code directions are indicated on the measure. The possible scores range from 15 to 105, and the subscale scores range from 5 to 35.

#### Emotion regulation questionnaire (ERQ)

The ERQ is a 10-item self-report scale to assess habitual use of two commonly used strategies for emotional regulation: cognitive reappraisal and expressive suppression (26). Responders answered each item with a 7-point Likert scale ranging from one (strongly disagree) to seven (strongly agree). Cognitive reappraisal involves thinking about a situation in a different perspective to change its meaning to alter one's emotional experience. Expressive suppression means a decrease in the outward expression of emotions. There are six items contributing to the subscale for cognitive reappraisal (e.g., “When I'm faced with a stressful situation, I make myself think about it in a way that helps me stay calm”) and four items contributing to the subscale for expressive suppression (e.g., “When I am feeling negative emotions, I make sure not to express them”).

#### Satisfaction with life scale (SWLS)

The SWLS is a 5-item scale designed to measure global cognitive judgments of one's life satisfaction (not a measure of either positive or negative affect) (27). Participants indicated their agreement/disagreement with each of the five items using a 7-point scale that ranges from seven (strongly agree) to one (strongly disagree).

#### Dissociative experiences scale (DES)

The DES is a 28-item self-report measure of dissociative experience. In the newer DES format, respondents circle a percentage, ranging from 0 to 100% at 10% intervals, indicating their agreement with the question. The DES score is the average of all questions; therefore, the minimum score is 0 and the maximum score is 100. All the questions are scored by dropping the zero on the percentage of each answer, e.g., 30% = 3; 80% = 8, these numbers are then added up. Scores of 30 or higher indicate high levels of dissociation.

#### The anxiety sensitivity index-3 (ASI-3)

The ASI-3 is an 18-item self-report measure developed to assess anxiety sensitivity (28). Each item is rated on a 5-point Likert scale ranging from zero (“not at all”) to four (“very much”); the higher the score, the more severe the anxiety sensitivity.

#### Positive emotion in distress scale (PEIDS)

The PEIDS is a 10-item Japanese self-report scale that assesses positive emotions during negative affective states, including broaden-and-build theory (29). Participants were asked to read each item and indicate the extent of their agreement or disagreement with 10 statements. Items were scored on a five-point Likert scale (1 = strongly disagree, 2 = disagree, 3 = neither agree nor disagree, 4 = agree, and 5 = strongly agree).

#### Affective style questionnaire (ASQ)

The ASQ is a 20-item scale used to assess emotion regulation in terms of three affective styles: concealing, adjusting, and tolerating (30). The items were measured using a five-point Likert scale.

### Outcome at time 2: Suicidal risk

#### Suicidal ideation attributes scale (SIDAS)

The SIDAS was used assesses the severity of suicidal ideation over the preceding month. There are five items asking the frequency, controllability, closeness to suicide attempt, level of distress associated with suicidal thoughts, and impact on daily function (31). Answers are responded with an 11-point Likert scale. The SIDAS was assessed both at Time 1 and Time 2. Responders with SIDAS scores of 21 or higher were regarded as having a risk of suicide (31). In the present study, we used SIDAS score at time 1 as a covariate and SIDAS score at time 2 as the outcome in the prediction model.

### Statistical analysis

#### Sensitivity analysis

We used Student's *t*-tests and chi-square tests to compare the characteristics between those who were lost to follow-up and participants at Time 2.

#### Model building and validation

We used Super Learner to develop the suicidality risk prediction model. Super Learner is an ensemble algorithm that uses a stacking process to determine the optimal weighted combination of a set of candidate algorithms using cross-validation to minimize the value of loss function (32). The values of weighted and loss function are considered the coefficient and risk. Super Learner can include many diverse algorithms and perform equally or better than the best-performing candidate algorithms. In process, we divided the data randomly into two sets: 70% into a training set and 30% into a test set. We estimated the risk of each algorithm using a 10-fold cross-validation. Super Learner combined all the candidate algorithms to generate a new algorithm with the best performance. All analyses were conducted using R version 4.2 (https://cran.r-project.org) and Super Learner 2.0–28 to develop the prediction models. In this study, we used 20 candidate algorithms to generate SuperLearners, including generalized linear mode, Bayesian generalized linear models, general additive model, five elastic-net regularized generalized linear models with alpha from zero to one with an increment of 0.25, kernel k nearest neighbors, support vector machine, linear discriminant analysis, neural networks, multivariate adaptive polynomial spline regression, random forests, and six extreme gradient boosting models by a grid of shrinkage parameter (0.1 and 0.01) with the number of terminal nodes (1, 2, and 4) (32). We found the performance of Super Learner is better than that of any specific algorithm (Supplementary Material I). The risk and coefficients are shown in Table 2. We then evaluated the performance of the model using a test dataset. The indicators of model performance included the AUC, sensitivity, specificity, and accuracy.

**Table 2:** Relative importance of the 10 top factors based on the suicide prediction model using measurements collected from the Time 1 responses of National Survey of Stress and Health.

	**%IncMSE**
SIDAS	5.85E−03
SDS	3.26E−03
PHQ-9	3.19E−03
CCSM-suicidal	2.69E−03
DES score	2.67E−03
Past history of self-harm	2.22E−03
GAD-7	1.97E−03
PCL-5	1.78E−03
CCSM-dissociation	1.76E-03
IES-R	1.66E−03

*SIDAS, suicidal ideation attributes scale; SDS, Sheehan disability scale; PHQ-9, Patient health questionnaire-9; CCSM, cross-cutting symptom measure for DSM-5; DES, Dissociative Experiences Scale; GAD-7, Generalized anxiety disorder 7-item scale; PCL-5, The PTSD Checklist for DSM-5; IES-R, Impact of event scale-revised*.

#### Identifying the top 10 risk factors

Given that Super Learner is a black box model, we used the random forest algorithm to train a model for the predicted value from Super Learner and to identify the variable importance measures for each predicator by calculating the increase in mean-squared errors, which indicated a decrease in accuracy after permutation of a predictor. The top 10 important risk factors were identified in this study. Furthermore, to address the problem of collinearity of the included variables, we measured the co-linearity using variable inflation factors (VIF). If the VIF ≥10, it indicated there is serious collinearity requiring correction. The results showed that the maximum of the VIFs of the variables is 6.038. Therefore, the possibility of collinearity of the included variables is less likely.

## Results

### Clinical characteristics of the study population

The baseline characteristics of study participants are shown in Table 1. Data from a total 3,090 respondents were analyzed (mean age, 44.9 ± 10.9 years; 48.8% female) at Time 1 of Wave 1, and 2,163 participants completed the survey at Time 2. There were no significant differences in the demographic characteristics between those who were lost to follow-up and those who remained in the study at Time 2. Among the responders, the most common traumatic experience was emotional abuse (60.7%, *n* = 1,875), followed by physical violence (39.6%, *n* = 1 222). The most common psychiatric comorbidity was MDD (52.8%), followed by panic disorder (20.4%), seasonal affective disorder (15.8%), and generalized anxiety disorder (15.8%).

### Performance of the suicide prediction model

**Figure 1:**
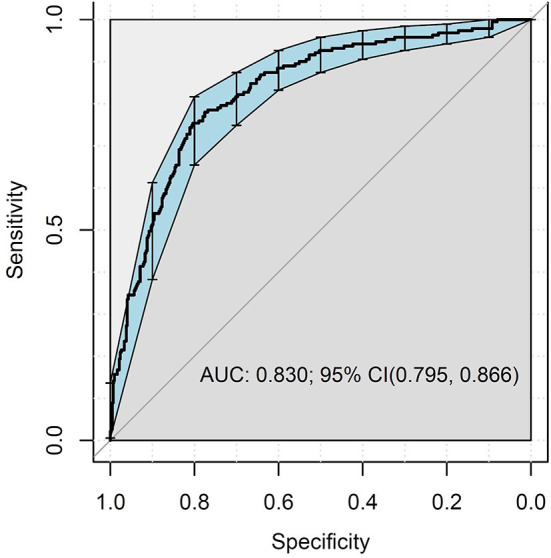
Area under the receiver operating curve of the predictive models of increased suicide risk.

A total of 65 factors were included as features to build the model. The model trained with 65 features showed a good performance (AUC = 0.830, 95% confidence interval [CI] = 0.795–0.866) in predicting future suicide risk (Figure 1). Overall, the model achieved an accuracy of 78.8%, sensitivity of 75.4%, specificity of 80.4%, positive predictive value (PPV) of 63.4%, and negative predictive value of 87.9%.

### Variable importance

The mean square error (%IncMSE) was used to evaluate variable importance in the model. Table 2 shows the 10 most important variables from the super-learner model. The most important risk factor was SIDAS score at Time 1. Other risk factors included the SDS score, PHQ-9 scores, CCSM-suicidal ideation domain, DES score, history of self-harm, GAD-7 score, PCL-5 score, CCSM-dissociation domain, and IES-R scores at baseline.

## Discussion

This is the first study to apply a machine-learning algorithm to online survey data to develop a model for predicting suicide risk in the general Japanese population. To our knowledge, few studies have integrated population-based datasets with machine-learning methods to predict suicide risk (10–12). The performance of our prediction model (AUC = 0.83, sensitivity = 75.4%, specificity = 80.4%) was similar to those previous studies using machine-learning approach in the general adult population in the United States (10) (AUC = 0.86, sensitivity = 85.3%, specificity = 73.3%) and South Korea (11) (AUC = 0.85, sensitivity = 83.6%, specificity = 80.7%), and much better than those using traditional methodology (AUC = 0.58) (2). Models for predicting suicidal outcomes that were developed in prior studies have been criticized for having low PPVs ( ≤ 50% in most models), which precluded their readiness for clinical applications in health care systems (33). Our model achieved a clinically actionable PPV (63.4%). These results are encouraging, given the recent emphasis on models in the general adult population using big data and their usefulness in developing precision treatment protocols for individuals at risk for suicide (8, 18).

One noteworthy finding in our study was that the most important risk factor in our prediction model was the baseline SIDAS. The SIDAS has proven to be a valid web-based measure for the severity of suicidal ideation. A previous study reported that scores ≥21 had a 95.8% specificity for the presence of a suicide plan in the past year and a 94.9% specificity for the presence of suicidal preparation/attempt in the past year (31). Our results indicated that SIDAS could be a good predictor of suicide risk.

Moreover, our results extend prior work by revealing the predictive value of variables related to functional impairment in three major life domains: work, social life/leisure activities, and family life/home responsibilities, as assessed by SDS, which are not covered in commonly used screening tools for suicide risk assessment. These findings may offer a new direction for improving suicidal behavior prediction through functional assessments.

Other important novel risk factors were related to emotional responses to traumatic experiences. The PCL-5, DES, and IES-R scores were moderate risk factors. The IES-R (34), PTSD symptoms (14, 15) and dissociative symptoms (35) are known risk factors for suicide in patients with traumatic experiences. Therefore, future assessment tools for suicide should include responders' past traumatic experiences and their related psychological consequences.

## Limitations

This study had several limitations. First, we employed an online self-report survey methodology to assess suicide risk and clinical and functional correlates. Although participation in the study was anonymous, the use of online surveys may increase the endorsement of sensitive responses due to increased anonymity (36). Furthermore, our results cannot be generalized to face-to-face interview assessments; however, our psychometric information may be useful for online epidemiological surveys or telemedicine. Second, we only included data from participants aged 18 years and older, and the risk factors identified might not be generalizable to children and adolescents. Third, we lacked information about suicide among participants lost to follow-up (i.e., Time 2 non-responders). Yet, the results of the sensitivity analysis showed that there was no significant difference in baseline demographic characteristics between those who were lost to follow-up and those under follow-up at Time 2. Fourth, the participants were limited to those who had Internet access and were registered as panelists for the survey company. To be specific, our study sample was relatively young and had lower personal income than the general Japanese adult population. The generalizability of these findings to other population remains unclear.

## Conclusions

Our study demonstrated the usefulness of machine learning methods to generate powerful suicide prediction models in a longitudinal cohort. We confirmed several well-known risk factors for suicide, such as the SIDAS and PHQ-9, while identifying new important risks. Specifically, functional impairment and emotional distress related to traumatic experiences emerged as novel, important factors in suicidal behavior. We hope that these results deepen our understanding of the etiology of suicide in adults and improve suicide prediction by identifying new risk variables to guide the future development of suicide risk assessment tools.

## Data availability statement

The raw data supporting the conclusions of this article will be made available by the authors, without undue reservation.

## Ethics statement

The studies involving human participants were reviewed and approved by National Center of Neurology and Psychiatry. The patients/participants provided their written informed consent to participate in this study.

## Author contributions

C-SW analyzed the data. P-HC and S-CW drafted the manuscript. MI conceived and designed the study, managed study administration, and including the ethical review process. MH and MI provided critical comments on the manuscript related to intellectual content. All authors contributed to the article and approved the submitted version.

## Funding

This study was supported by a Grant-in-Aid for Scientific Research (A) (15H01979), awarded to MH, from the Japan Society for the Promotion of Science, Tokyo, Japan. The funders had no role in the design and conduct of the study; collection, management, analysis, and interpretation of the data; preparation, review, or approval of the manuscript; and decision to submit the manuscript for publication.

## Conflict of interest

The authors declare that the research was conducted in the absence of any commercial or financial relationships that could be construed as a potential conflict of interest.

## Publisher's note

All claims expressed in this article are solely those of the authors and do not necessarily represent those of their affiliated organizations, or those of the publisher, the editors and the reviewers. Any product that may be evaluated in this article, or claim that may be made by its manufacturer, is not guaranteed or endorsed by the publisher.
