# Supplementary material for: A machine-learning model to predict suicide risk in Japan based on national survey data
Source: Front Psychiatry. 2022 Aug 4;13:918667. doi: 10.3389/fpsyt.2022.918667 (PMC9387201; doi:10.3389/fpsyt.2022.918667)
Supplement: Supplementary file 1 [file Data_Sheet_1.DOCX]

**Table 1. The area under the receiver operating curve for the selected 20 algorithms.**

| SL.bayesglm | 0.794 (0.756, 0.831) |
| --- | --- |
| SL.gam | 0.790 (0.752, 0.829) |
| SL.glm | 0.793 (0.756, 0.831) |
| SL.glmnet (alpha=0) | 0.819 (0.783, 0.854) |
| SL.glmnet (alpha=0.25) | 0.819 (0.784, 0.855) |
| SL.glmnet (alpha=0.5) | 0.820 (0.785, 0.855) |
| SL.glmnet (alpha=0.75) | 0.818 (0.782, 0.854) |
| SL.glmnet (alpha=1) | 0.820 (0.784, 0.855) |
| SL.kernelKnn | 0.749 (0.708, 0.789) |
| SL.ksvm | 0.800 (0.763, 0.837) |
| SL.lda | 0.796 (0.758, 0.834) |
| SL.nnet | 0.685 (0.647, 0.724) |
| SL.polymars | 0.799 (0.762, 0.836) |
| SL.ranger | 0.804 (0.767, 0.841) |
| SL.xgboost (max_depth=1, shrinkage=0.01) | 0.806 (0.769, 0.842) |
| SL.xgboost (max_depth=1, shrinkage=0.1) | 0.786 (0.748, 0.824) |
| SL.xgboost (max_depth=2, shrinkage=0.01) | 0.776 (0.737, 0.815) |
| SL.xgboost (max_depth=2, shrinkage=0.1) | 0.817 (0.781, 0.852) |
| SL.xgboost (max_depth=4, shrinkage=0.01) | 0.820 (0.784, 0.855) |
| SL.xgboost (max_depth=4, shrinkage=0.1) | 0.814 (0.778, 0.850) |

**Abbreviations**: glm, generalized linear mode; bayesglm, Bayesian generalized linear model; gam, general additive model; glmnet, five elastic-net regularized generalized linear models with alpha from 0 to 1 with an increment of 0.25; kernelKnn, kernel k nearest neighbors; ksm, support vector machine; lda, linear discriminant analysis; nnet, neural networks; polymars, multivariate adaptive polynomial spline regression; ranger, random forests; xgboost, six extreme gradient boosting model.
